# Controlled Synthesis and Properties of 3*d*–4*f* Metals Co-doped Polyoxometalates-Based Materials

**DOI:** 10.1186/s11671-020-03431-9

**Published:** 2020-11-04

**Authors:** Ning Liu, Ningning Guo, Lin Sun, Shixian Liu, Guan Wang, Yuan Zhao

**Affiliations:** 1 Henan Key Laboratory of Polyoxometalate Chemistry, Institute of Molecular and Crystal Engineering, College of Chemistry and Chemical Engineering, Henan University, Kaifeng, Henan China; 2 The Key Laboratory of Natural Medicine and Immuno-Engineering, Henan University, Kaifeng, Henan China

**Keywords:** Polyoxometalate, Morphology control, Photoluminescence, Magnetism

## Abstract

It is challenging to explore and prepare polyoxometalates-based nanomaterials (PNMs) with controllable morphologies and diversiform components. Herein, 3*d*–4*f* metals are introduced into isopolyoxometalates and Anderson-type polyoxometalates, CeCdW_12_ nanoflower and EuCrMo_6_ microflaky have been fabricated respectively. A series of control experiments are carried out to identify the impact factors on the rare morphologies in PNMs. Furthermore, upon excitation at 396 nm, the emission spectrum of EuCrMo_6_ displays five prominent *f − f* emitting peaks at 674, 685, 690, 707, and 734 nm that are assigned to Eu^3+ 5^D_0_ → ^7^F_J_ (*J* = 0, 1, 2, 3, 4) transitions. Meanwhile, the VSM results show that the Cr^+3^ ions in EuCrMo_6_ display anti-ferromagnetic interactions when the temperature is lower than − 17.54 K. After rising temperature, this material exhibits paramagnetic property. This work opens up strategies toward the brand new morphologies and components of PNMs, endowing this kind of material with new functions.

## Introduction

Due to the intriguing structures and diverse properties, POMs have a wide range of applications in catalysis, magnetism, medicine, and materials science [1–7]. As a special branch, PNMs have many advantages in contrast with traditional single-crystal compounds. For example, the size, morphology and chemical composition of PNMs can be easily tuned by modern nanosynthesis technology [8, 9]. Therefore, the research of PNMs have been gradually attracted much attention, and various PNMs with diverse morphologies and properties have been reported until now [10–12]. In 2012, Liu’s group found polyoxoanions with high solubility in water and/or other polar solvents demonstrate unique solution behavior by self-assembling into single layer, hollow, spherical blackberry structures [13]. After that, star-shaped Keggin-type heteropolytungstate was obtained as catalyst for preparing quinoline derivatives [14]. From then on, Chattopadhyay’s and co-workers discovered the Dexter-Silverton type molybdenum tungstate of hollow microspheres [15]. For the past years, ours group has been working on the control-synthesis and functionalization of POM-based nano/micromaterials by chemical precipitation or hydrothermal methods [16–18]. In particular, we found that the morphology and photoluminescence properties of CeF_3_ nanocrystals can be finely tuned by doping different amount/type of POMs [19].

POMs containing 3*d*–4*f* metals display remarkable magnetic, catalytic, and optical properties, which endow them with wide range of applications [20, 21]. For example, unprecedented structures based on monovacant POMs capped by heterometallic 3*d*–4*f* {LnCu_3_(OH)_3_O} (Ln = La, Gd, Eu) cubane fragments were characterized and their magnetic properties were also investigated [22]. Powell et al. addressed a giant 3*d*–4*f* tetrahedral heterometallic POM, which showed single-molecule magnet behavior in 2015 [23]. One year later, a series of organic–inorganic hybrid POMs constructed from 3*d*–4*f* heterometallic sandwiched polyoxotungstate dimers were isolated. X-ray single-crystal diffraction reveals that these compounds exhibited supramolecular nanotube structures [24].

It can be seen from these literatures that the study of 3*d*–4*f* POM mainly focuses on traditional single-crystal compounds, and the research on 3*d*–4*f* PNMs is still rare. Therefore, introducing 3*d*–4*f* metals into PNMs to synthesize new materials with novel morphologies and special properties has become one of our research objectives. Furthermore, most of the reported PNMs are based on Keggin type heteropolyoxometalates. Isopolyoxometalates and Anderson-type POMs are seldom used as building blocks to construct PNMs. From these perspectives, how to construct isopolyoxometalates or Anderson-type POM-based 3*d*–4*f* PNMs become the focus of our research. In this report, Na_2_WO_4_·2H_2_O, Na_2_MoO_4_·2H_2_O and other simple substances as starting materials were employed to synthesize 3*d*–4*f* PNMs. Fortunately, two novel 3*d*–4*f* PNMs named CeCdW_12_ and EuCrMo_6_ were obtained by chemical precipitation method. It is worth to note that these materials are built on isopolyoxometalates sodium paratungstate and Anderson type [CrMo_6_O_24_H_6_]^3–^, respectively. Moreover, CeCdW_12_ and EuCrMo_6_ exhibit uniform flower-like and flaky morphologies, which are both rarely found in PNM chemistry. These peculiar morphologies attract our interest and a series of control experiments are carried out to explore regular phenomena. Finally, according to the composition of these materials, photoluminescence and magnetic properties of CeCdW_12_ and EuCrMo_6_ are investigated. The strategy demonstrated in this work could be applied to prepare novel PNMs with various morphologies or compositions. Following, it could provide a potential method to separate multifunctional PNMs for optoelectronic devices, high-density magnetic memories and so on.

## Methods

All chemicals were reagent-grade and used without further purification. Na_6_[H_2_W_12_O_40_] was synthesized according to ref. 25 identified by IR spectrum. The XRD of CeCdW_12_ nanoflowers and EuCrMo_6_ microflakes were obtained on a Bruker D8 Advance instrument with Cu Kα radiation (*λ* = 1.5418 Å) in the 2*θ* range from 10° to 80° and 10° to 40°, respectively. The SEM image and EDX spectrum were identified by a JSM-7610F scanning electron microscopy with an acceleration voltage of 10 kV. IR spectra were recorded on an Avatar 360 Fourier transform infrared (FTIR) spectrophotometer using KBr pellets in the range of 4000–450 cm^−1^. The X-ray photoelectron spectra (XPS) were collected using a PHI 5000 VersaProbe (U1VAC-PHI). Inductively coupled plasma optical emission spectroscopy (ICP-AES) experiments were performed on a Perkin-Elmer Optima 2100DV optical emission spectrometer. Electrospray ionization mass spectrometry (ESI-MS) routine spectra were carried out with a Bruker MTQ III-QTOF. The experiments were performed with the negative ion mode in acetonitrile solvent by direct infusion with a syringe pump with a flow rate of 5 μL min^−1^. The PL spectra were collected by a Hitachi F-7000 fluorescence spectrophotometer. The PL lifetime was performed on an Edinburgh Instruments FLS980 spectrophotometer.

### Synthesis of CeCdW_12_ Nanoflowers

Na_2_WO_4_·2H_2_O (3.00 g, 9.10 mmol) was dissolved in 30 mL of distilled water, the solution was heated to 80 °C, stirred and boric acid (0.10 g, 1.62 mmol) was added to the solution. And then, the system pH was adjusted to 7 with dilute HCl. After that, a small amount of an aqueous solution containing CdCl_2_·2.5H_2_O (0.46 g, 2.00 mmol) and Ce(NO_3_)_3_·6H_2_O (0.87 g, 2.00 mmol) was slowly added dropwise, and if a precipitate formed, it was completely dissolved and then added to the next drop. After the completion of the dropwise addition, the system pH was adjusted to 6 with dilute HCl. Stirring was continued at this temperature for another half an hour. Finally, saturated KCl solution was added dropwise in order to form light yellow precipitation. Then, CeCdW_12_ nanoflowers was collected by centrifuge and washed with water and ethanol to removed excess regents.

### Synthesis of Na_3_[CrMo_6_O_24_H_6_]·8H_2_O

Na_3_[CrMo_6_O_24_H_6_]·8H_2_O was prepared according to the previous literature [26]. In the typical method, Na_2_MoO_4_·2H_2_O (14.50 g, 0.06 mol) was dissolved in 30 mL distilled water and the pH was adjusted to 4.5. Then 4 mL of solution containing Cr(NO_3_)_3_·9H_2_O (4.00 g, 0.01 mol) was added and the mixture was boiled for 1 min. Following, the solution was filtered while hot, next, saturated KCl solution was dripped into the filtrate slowly to give precipitate. Finally, the solid product was collected by centrifuge and washed with water and ethanol to removed excess regents.

### Synthesis of EuCrMo_6_ Microflakes

Na_3_[CrMo_6_O_24_H_6_]·8H_2_O (0.12 g, 0.10 mmol) was dissolved in 20 mL distilled water. The solution was heated to 60 °C, and 5 mL solution containing Eu(NO_3_)_3_·6H_2_O (0.09 g, 0.20 mmol) was added dropwise. The mixed solution was heated at 60 °C for another 40 min and filtered after cooling to room temperature. Take the filtrate and add NH_4_Cl solution (6.92 mol/L) was dropwise to give the precipitate. Then, the homogeneous mixture was stirred for another 6 h. Finally, the white solid product of EuCrMo_6_ microflakes was collected by centrifuge and washed with water and ethanol to removed excess regents.

## Results and Discussion

**Scheme 1:**
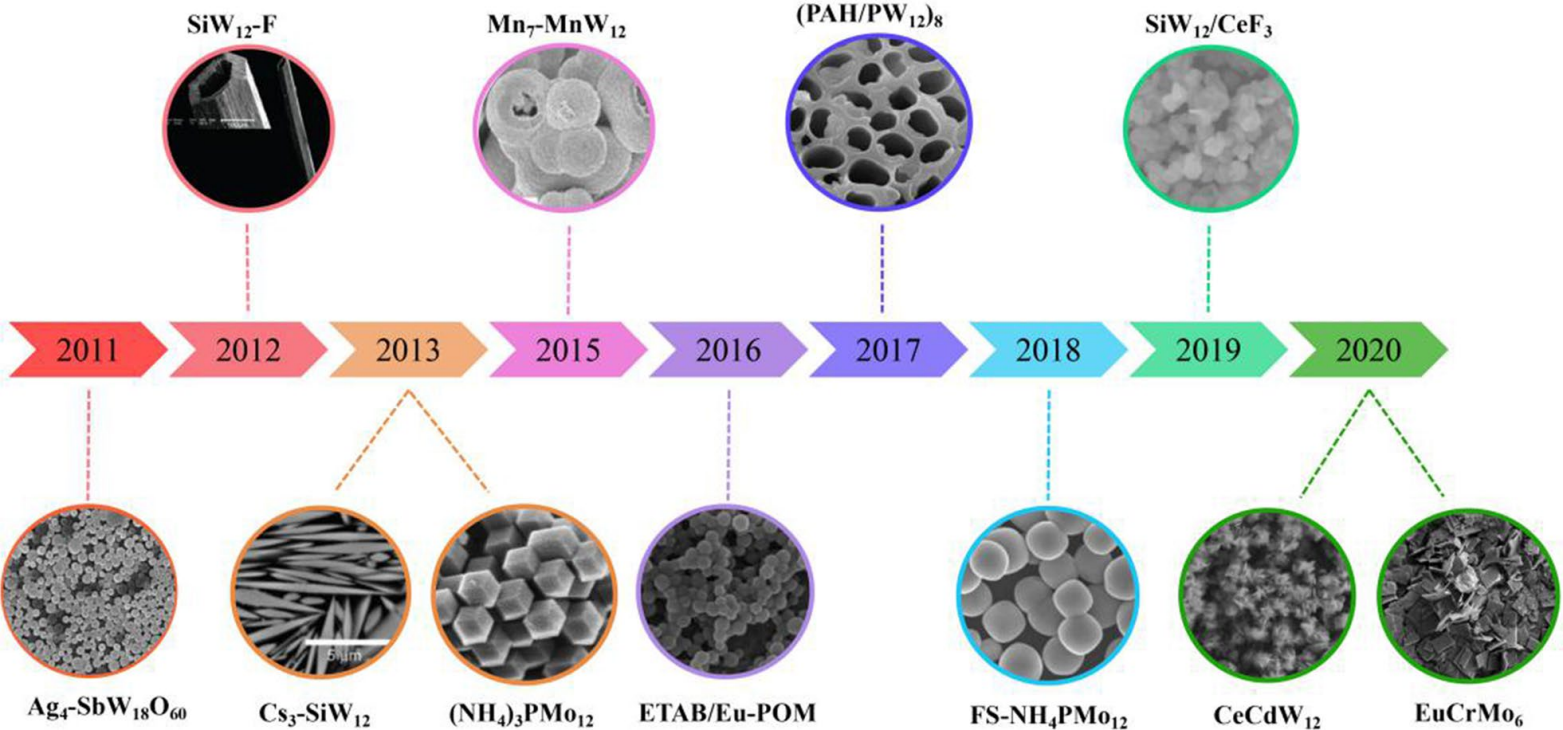
Summary of some typical micro- or nanomorphologies of POM from 2011 to 2020

**Scheme 2:**
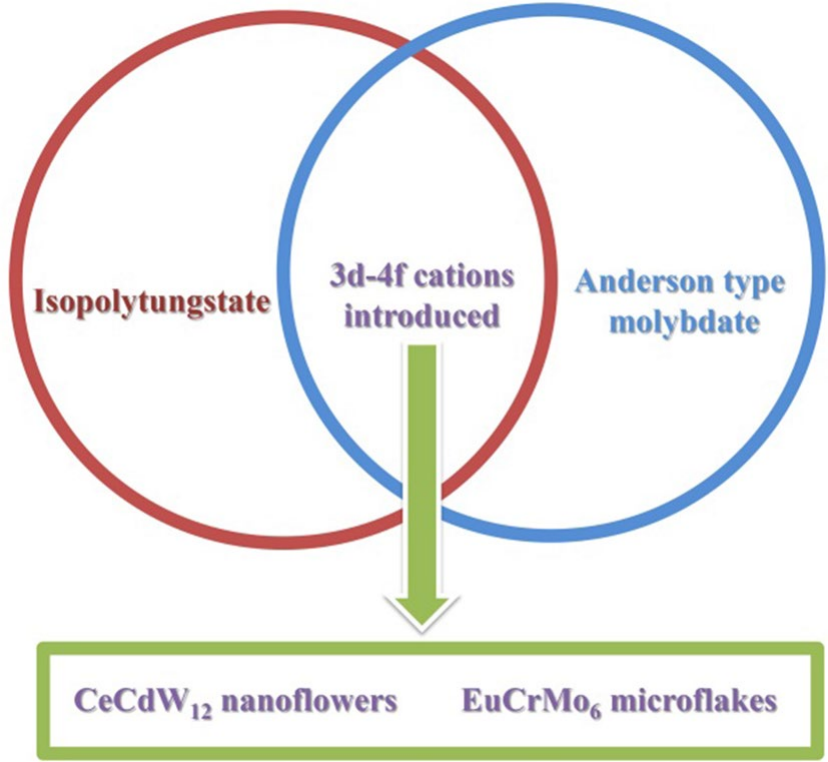
3*d*–4*f* cations introduced CeCdW_12_ nanoflowers and EuCrMo_6_ microflakes

**Scheme 3:**
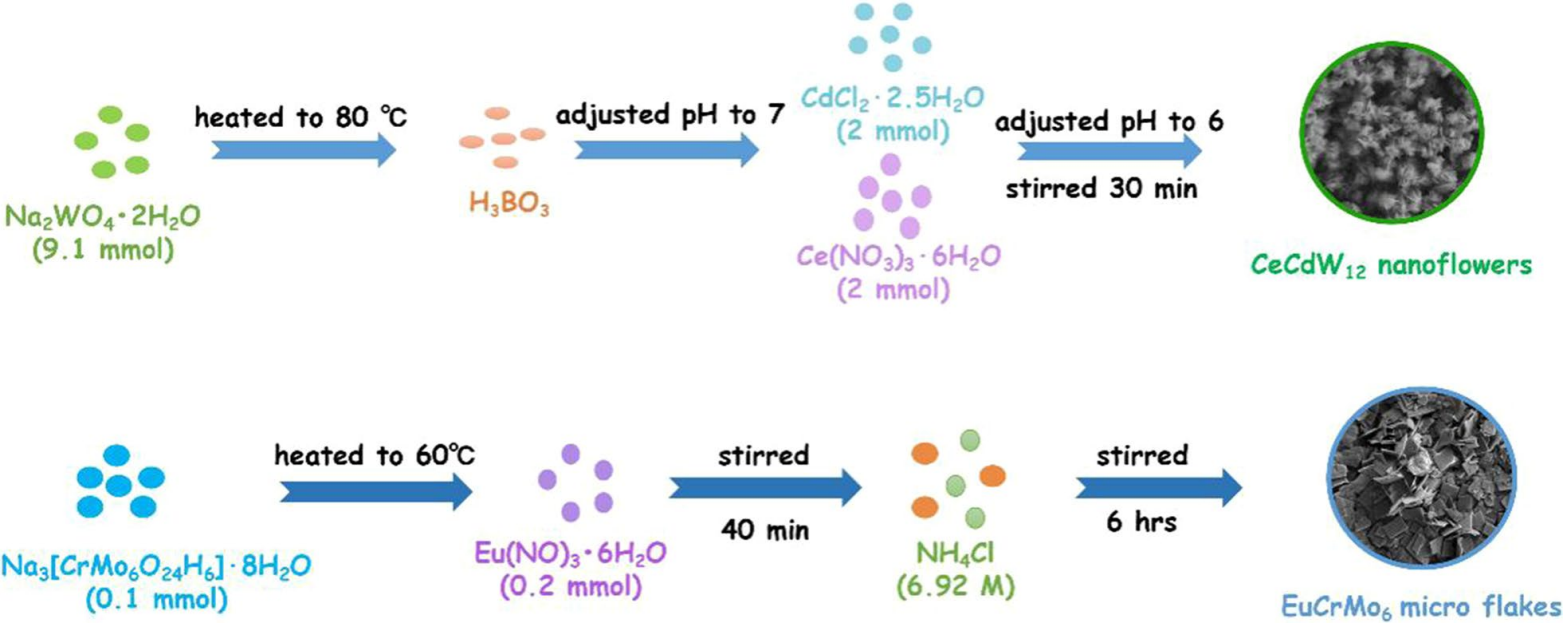
Synthetic strategy of two 3*d*–4*f* metals doped PNMs

In the past 10 years, due to the excellent properties, POM-based nano/micromaterials have been attracted wide attention in various fields. Numerous materials have been addressed with different morphologies (Scheme 1). However, compared to traditional single-crystal POM compounds, there are many merits problem need to depth study. On the one hand, the building blocks of PNMs are almost saturate Keggin type POMs. Many other POMs are seldom used to prepare PNMs, such as Anderson type, Waugh type, Silverton type, Dawson type, Standberg type and Weakely type. On the other hand, isopolyoxometalates are also rarely employed as starting materials or building blocks to introduce into PNMs. Finally, the reported PNMs are only containing transition metals, rare-earth ions are rarely used. Based on these perspectives, we used the isopolytungstate and Anderson type molybdate which were seldom employed to combine with 3*d*–4*f* cations in this work (Scheme 2). Fortunately, two new PNMs with novel morphologies have been isolated by using chemical precipitation method (Scheme 3), and their fluorescence and magnetism properties have been also investigated in this paper.

**Fig. 1:**
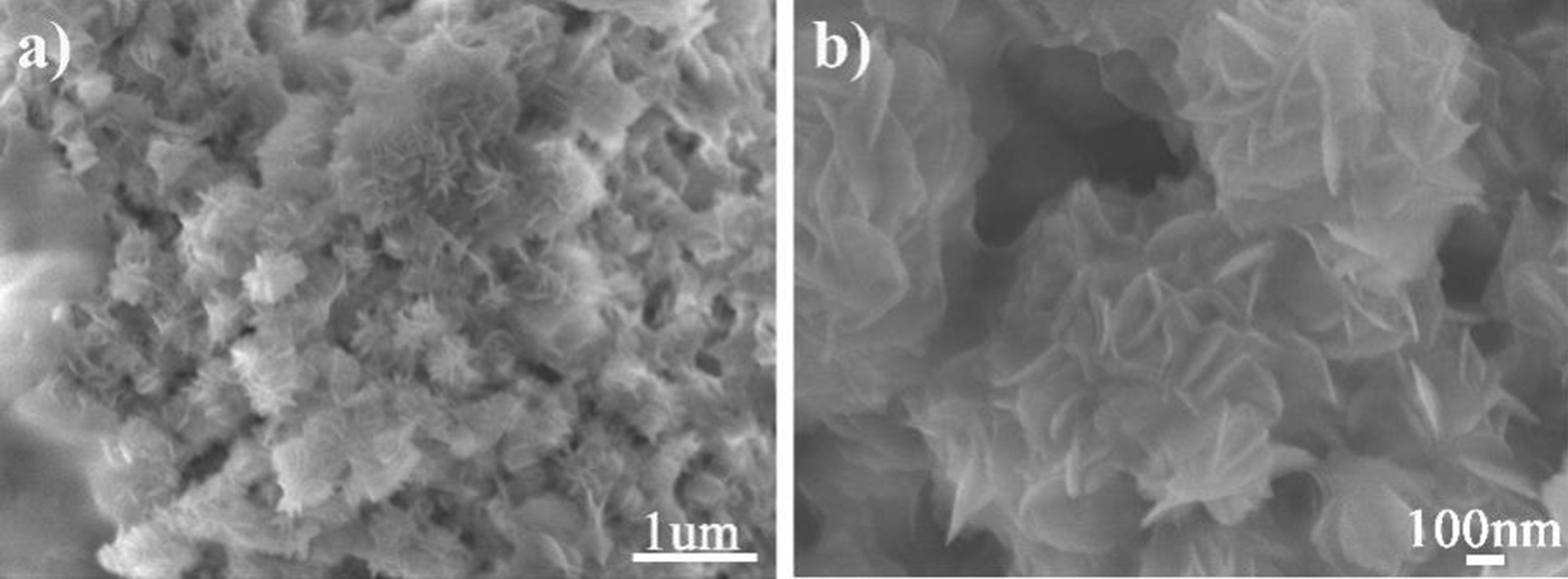
SEM images of CdW_12_ nanoflowers

At the beginning of this work, the different morphologies which have been formed during the experimental process raised our concerns. These phenomena might be impacted by different synthetic procedures. In order to figure out the impact factors of the morphologies, a series of control experiments have been carried out. CeCdW_12_ nanoflowers have been taken for example. First of all, considering the influence of the rare earth metals on the morphology of products, only Cd^2+^ cations were utilized, under the same conditions. Beyond our expectation, CdW_12_ nanoflowers were obtained (Fig. 1), from which it could be seen it is composed of flower-like morphology in nanosize. Thus, these evidences indicate that the absence of Ce^3+^ cations does not affect the morphology of this material. On the contrary, Cd^2+^ cations may play an important role in the formation of flower-like morphology.

**Fig. 2:**
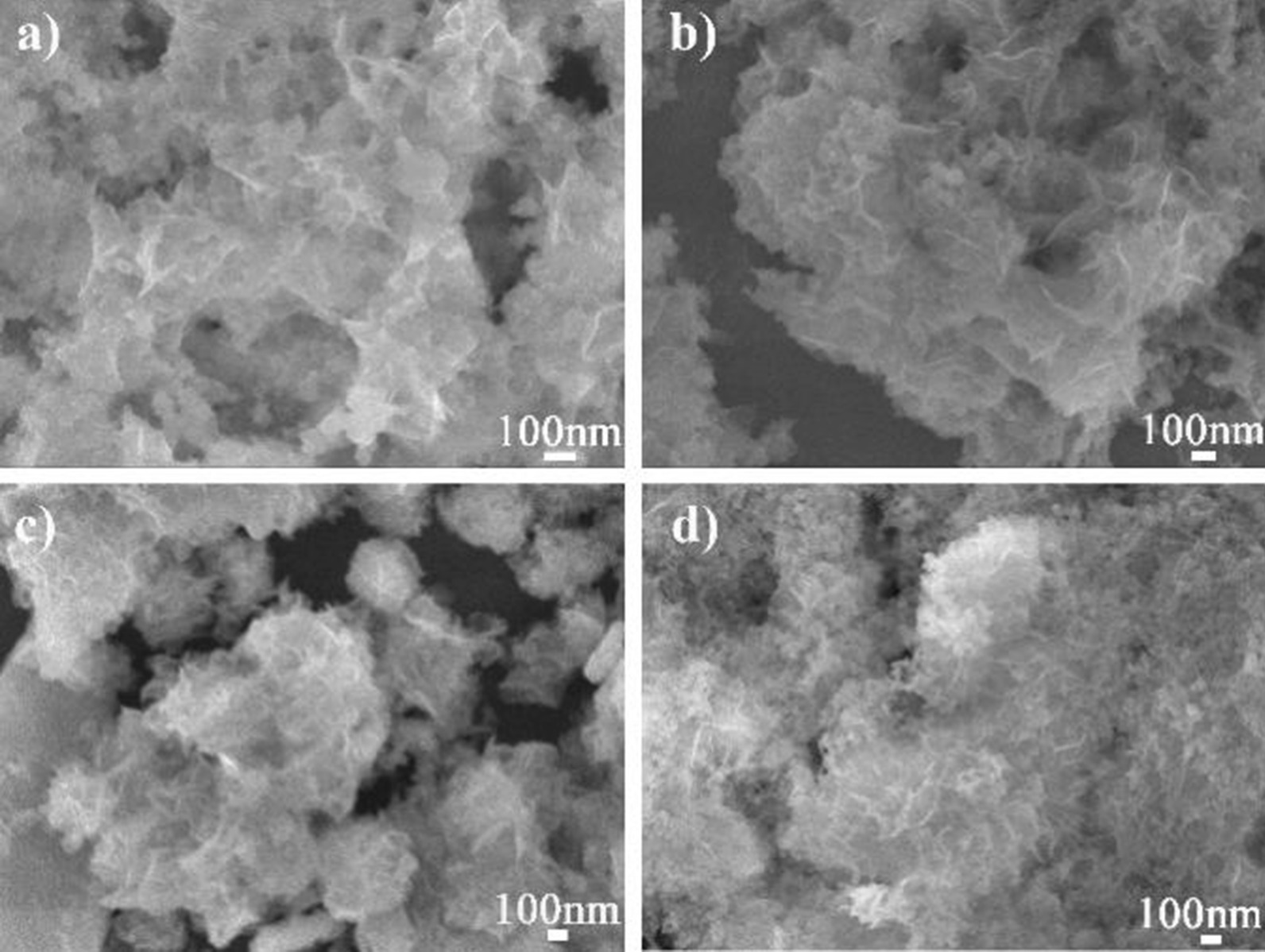
SEM images of CeCdW_12_ nanoflowers which were prepared using different amount of CdCl_2_·2.5H_2_O (**a** 0.5 mmol; **b** 1.0 mmol; **c** 3 mmol; **d** 3.5 mmol)

In this case, other control experiments were carried out to explore this system. Under similar approach to CeCdW_12_ nanoflowers, only the amount of CdCl_2_·2.5H_2_O was changed from 0.5 to 3.5 mmol. As depicted in Fig. 2, the SEM images exhibit different results obviously. When the dosage of CdCl_2_·2.5H_2_O were less than 2 mmol, porous bulks were formed. However, these architectures were not continued to evolve to nanoflowers. Furthermore, when the usage of CdCl_2_·2.5H_2_O were increased to more than 3 mmol, different situations were observed. Although monodispersed nanoflowers were prepared, abundant amorphous powders were appeared simultaneously. Therefore, these evidences prove that appropriate amounts of Cd^2+^ cations would help this material to assemble into nanoflower morphology. Otherwise, the self-aggregation of the novel morphology could be obstructed under excess Cd^2+^ cations.

**Fig. 3:**
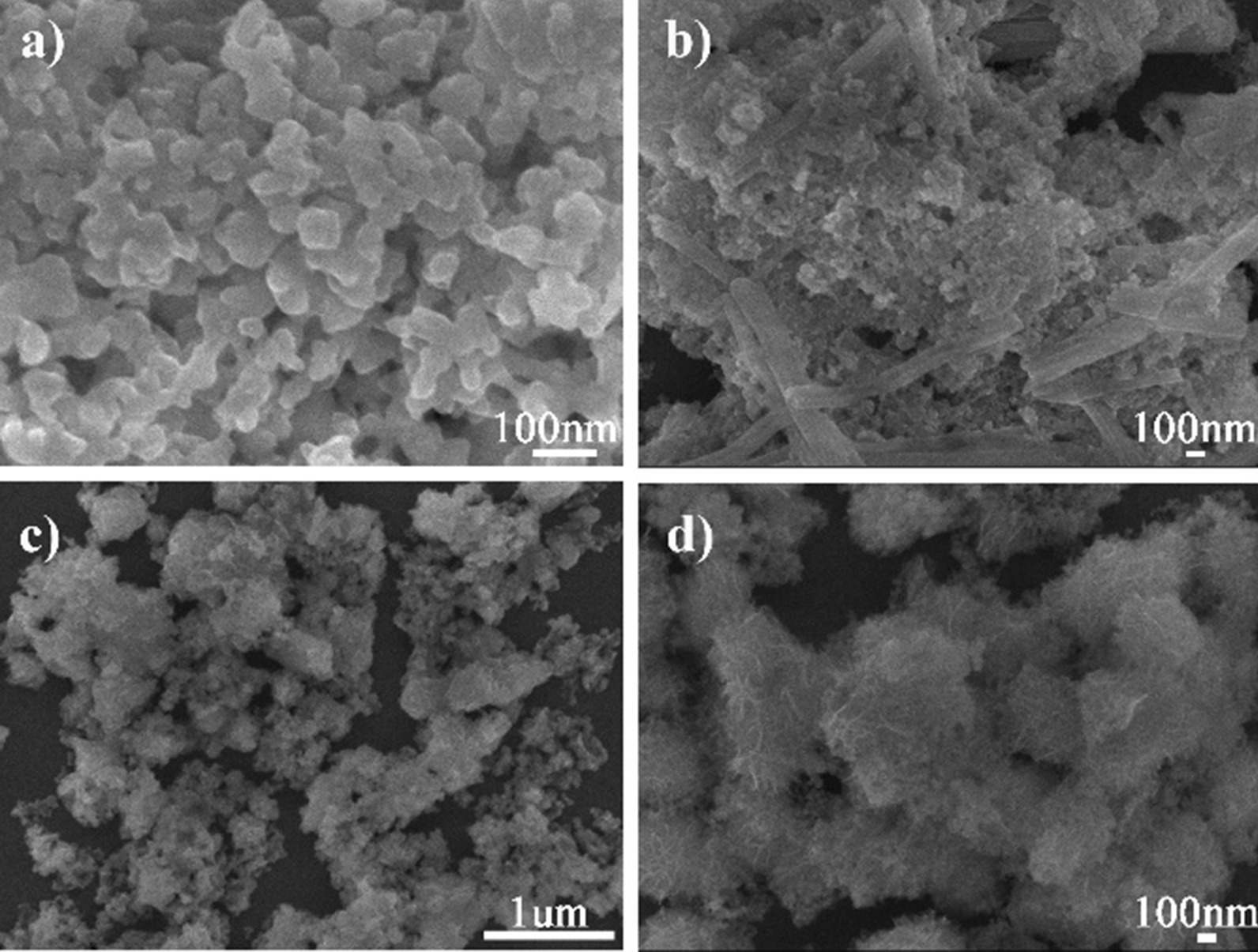
SEM images of CeCdW_12_ nanoflowers which prepared under different pH values (the pH values from **a** to **d** is 2, 3, 4 and 7, respectively)

Suitable pH value might be an important condition for the crystallization of CeCdW_12_ nanoflowers. In order to verify these hypotheses, the other control experiments were tried out. Under the methods which were similar to CeCdW_12_ nanoflowers, the pH values were adjusted to 2, 3, 4 and 7 before adding precipitant KCl. The results are shown in Fig. 3, the morphologies of CeCdW_12_ are changed apparently. When the pH values are lower than 5, irregular shapes could be observed, even some nanorods are observed in Fig. 3b. With the increase in pH value, flower-like morphology could be formed. These evidences indicate that strong acid condition is not suitable for the growth of CeCdW_12_ nanoflowers.

### IR Spectra

**Fig. 4:**
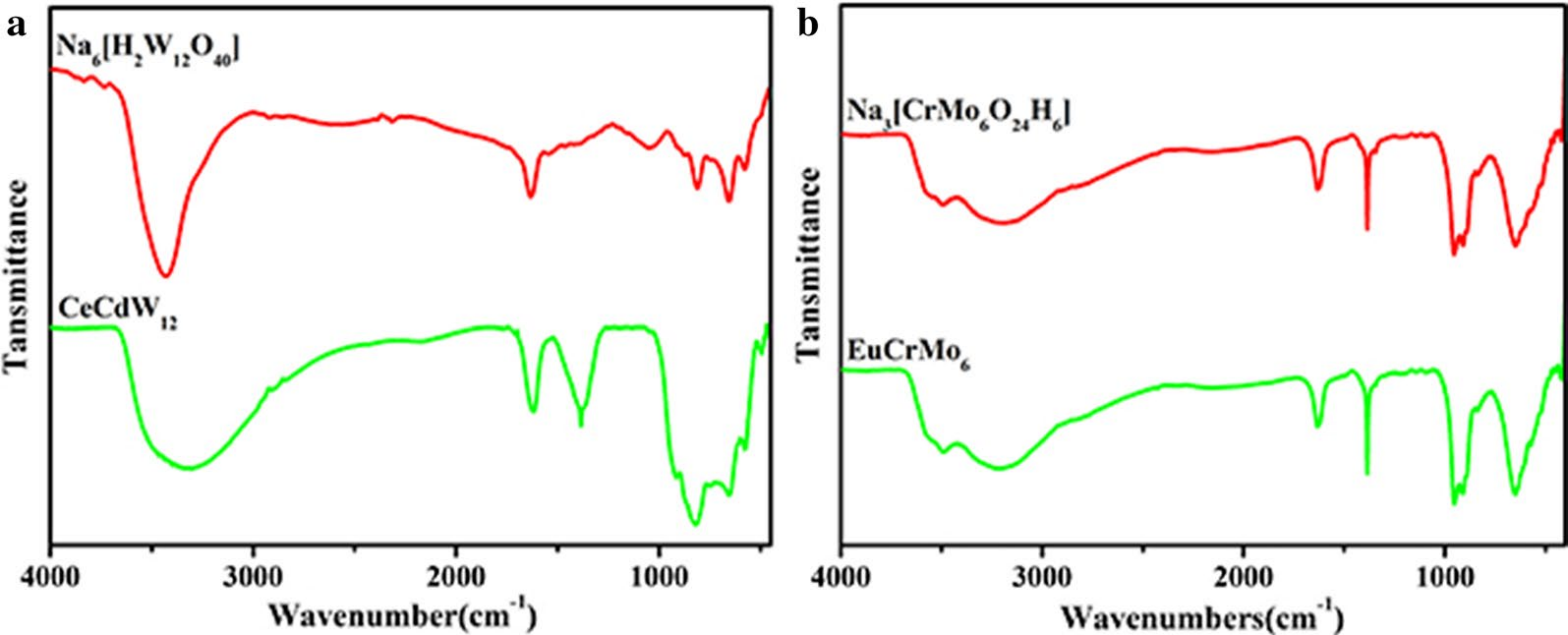
**a** IR spectra of CeCdW_12_ nanoflowers and **b** EuCrMo_6_ microflakes

IR spectra of sodium metatungstate Na_6_[H_2_W_12_O_40_] (refer as ‘W_12_’ for short), CeCdW_12_ nanoflowers, Na_3_[CrMo_6_O_24_H_6_] (refer as ‘CrMo_6_’ for short) and EuCrMo_6_ microflakes were recorded between 450 and 4000 cm^−1^ with KBr pellet (Fig. 4a), which is very useful for the identification of characteristic vibration bands of POMs in products. Firstly, IR spectrum of CeCdW_12_ nanoflowers exhibits characteristic vibration absorption bands of the metatungstate polyoxoanion. The bands at 654 cm^−1^, 823 cm^−1^ and 917 cm^−1^ for CeCdW_12_ nanoflowers are attributed to the vibration of the *ν*(W–O) bonds [25]. Secondly, IR spectra of Na_3_[CrMo_6_O_24_H_6_] and EuCrMo_6_ microflakes were also observed between 450 and 4000 cm^−1^ (Fig. 4b). The EuCrMo_6_ microflakes could be identified by two strong characteristic IR bands appearing at 1086 cm^−1^ (Cr–O), 904 cm^−1^ (Mo = O) and 834 cm^−1^ (Mo-O_b_-Mo), which is in accordance with the bulk Na_3_[CrMo_6_O_24_H_6_] [27]. These results indicate the building blocks of CeCdW_12_ nanoflowers and EuCrMo_6_ microflakes are isopolyoxometalates [H_2_W_12_O_40_]^6–^ and Anderson type [CrMo_6_O_24_H_6_]^3–^, respectively.

### XRD Patterns

**Fig. 5:**
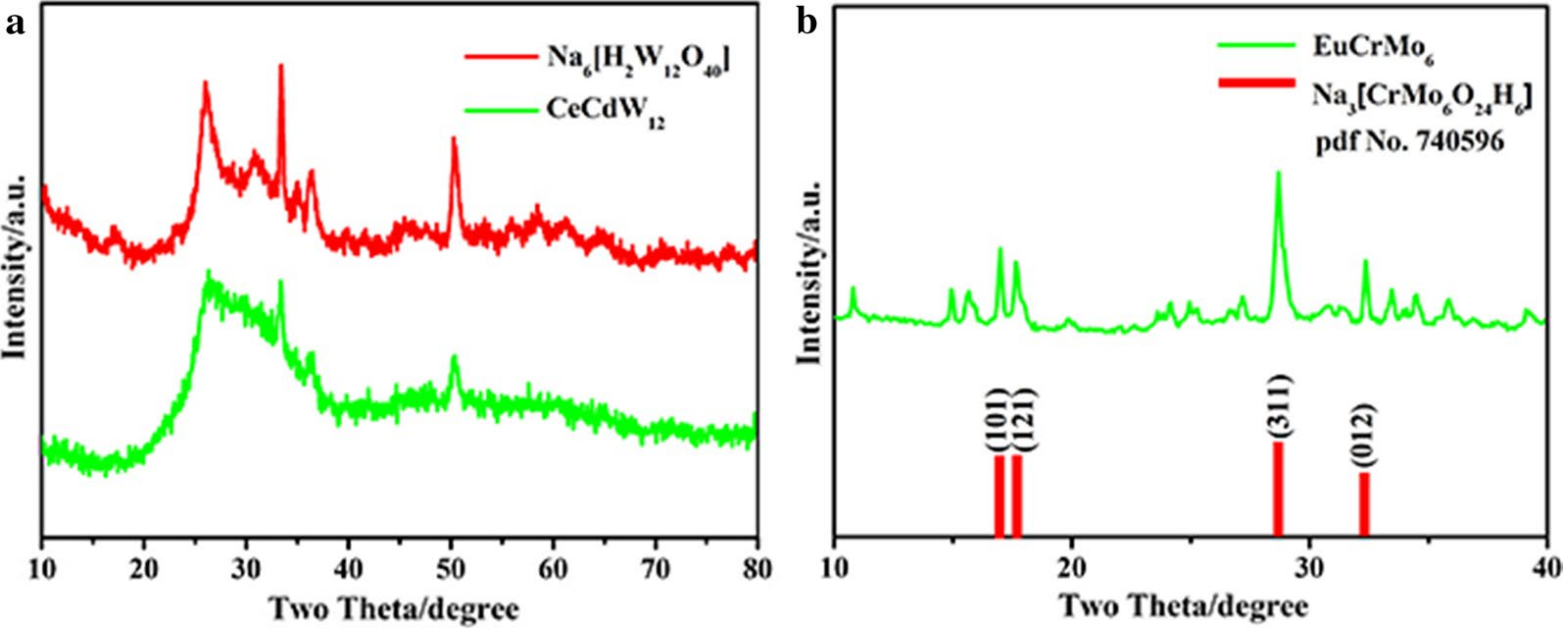
XRD patterns of CeCdW_12_ nanoflowers and EuCrMo_6_ microflakes

The as-prepared CeCdW_12_ nanoflowers, EuCrMo_6_ microflakes and their precursors were characterized by XRD. As can be seen from Fig. 5a, the main peaks of CeCdW_12_ nanoflowers at 25.9°, 33.2°, 36.3° and 50.3° in the range of 20°–55° can be readily indexed to the sodium metatungstate Na_6_[H_2_W_12_O_40_]. The results reveal that the CeCdW_12_ nanoflowers are constructed from metatungstate polyanions. In addition, the main peaks of EuCrMo_6_ microflakes at 17.0°, 17.6°, 28.7° and 32.4° can be readily indexed to the Na_3_[CrMo_6_O_24_H_6_] (Fig. 5b). According to the standard cards of Na_3_[CrMo_6_O_24_H_6_]·8H_2_O (pdf no. 740596), EuCrMo_6_ microflakes exhibit primitive structure and the above-mentioned 2*θ* peaks are attributed to (101), (121), (311) and (012) crystal planes, respectively. The results reveal that the structure of Anderson-type POM is preserved in the final product.

### SEM Images

**Fig. 6:**
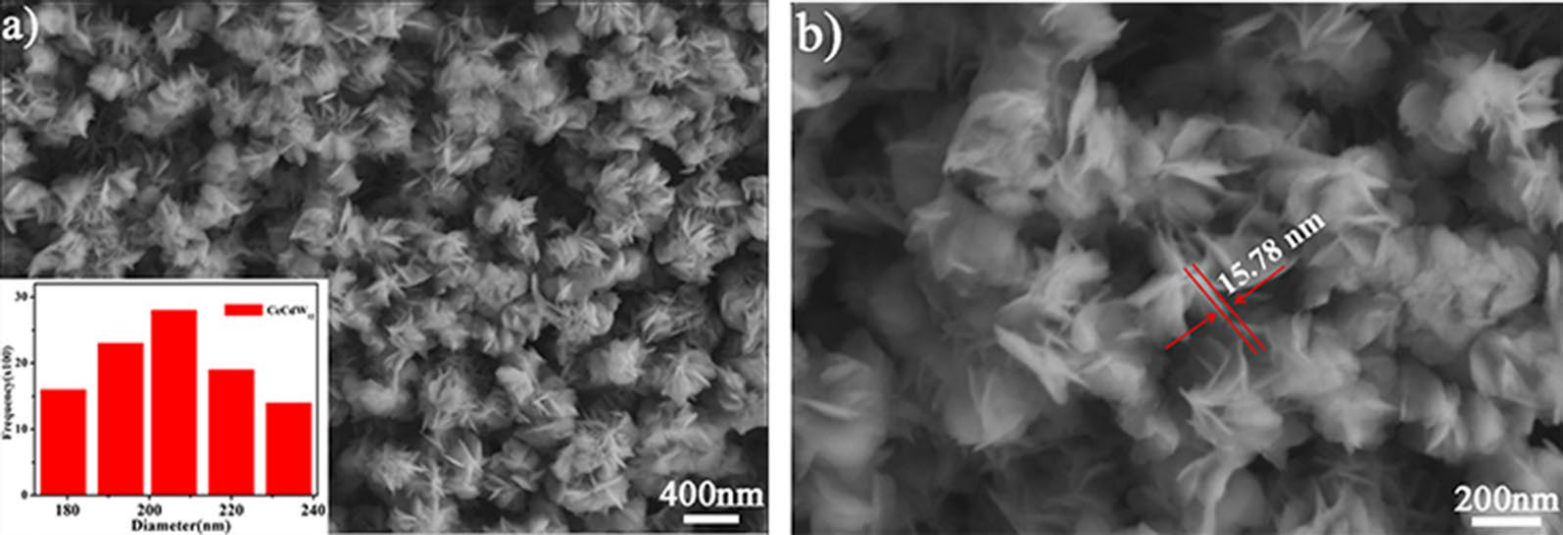
SEM images of CeCdW_12_ nanoflowers (inset: size distribution)

Figure 6 shows a typical SEM micrograph of CeCdW_12_ nanoflowers which are characterized using silicon wafer as a substrate. As can be seen from the images, this material exhibit uniform and monodisperse nanoflower morphology. According to the statistical 100 particles, the average diameter of these nanoflowers is about 177 nm. Under high resolution observation, the thickness of the nanosheet is ca. 15.78 nm. To the best of our knowledge, this kind of peculiar morphology is quite rare in the research filed of PNMs. Last year, CeF_3_ nanoflowers have been prepared by using POMs as dopants in our group. Interestingly, the CeCdW_12_ nanoflowers are very different from our previous work. Firstly, the particle size of CeCdW_12_ nanoflowers (177 nm) is much smaller than POM/CeF_3_ (630 nm). Secondly, CeCdW_12_ nanoflowers are built by almost disordered nanosheets rather than orderly stacking. Finally, the major component of CeCdW_12_ nanoflowers is POM, this is also markedly different from the nanoflowers of rare earth fluorides.

**Fig. 7:**
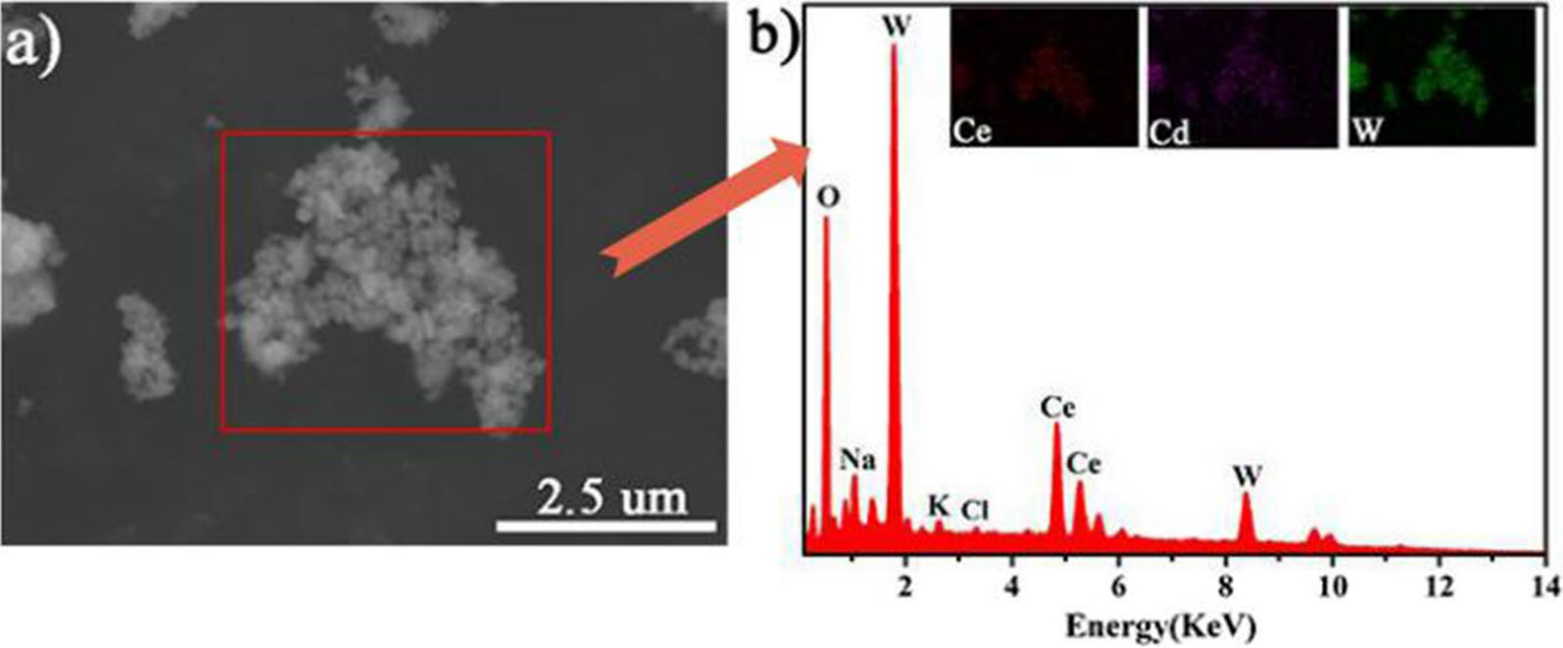
Corresponding element mappings and EDX of CeCdW_12_ nanoflowers

In order to identify the components of the CeCdW_12_ nanoflowers, the corresponding element mappings and EDX were investigated (Fig. 7). In these tests, the sample was prepared using silicon wafer as a substrate. The analyses evidently prove the presence of Ce, Cd and W components and the content of tungstate is much more than 3*d*–4*f* metals. Meanwhile, the element mappings of Ce and Cd show homogeneous distribution in this nanocomposite, indicating the chemical precipitation process is suitable for doping two different metals.

**Fig. 8:**
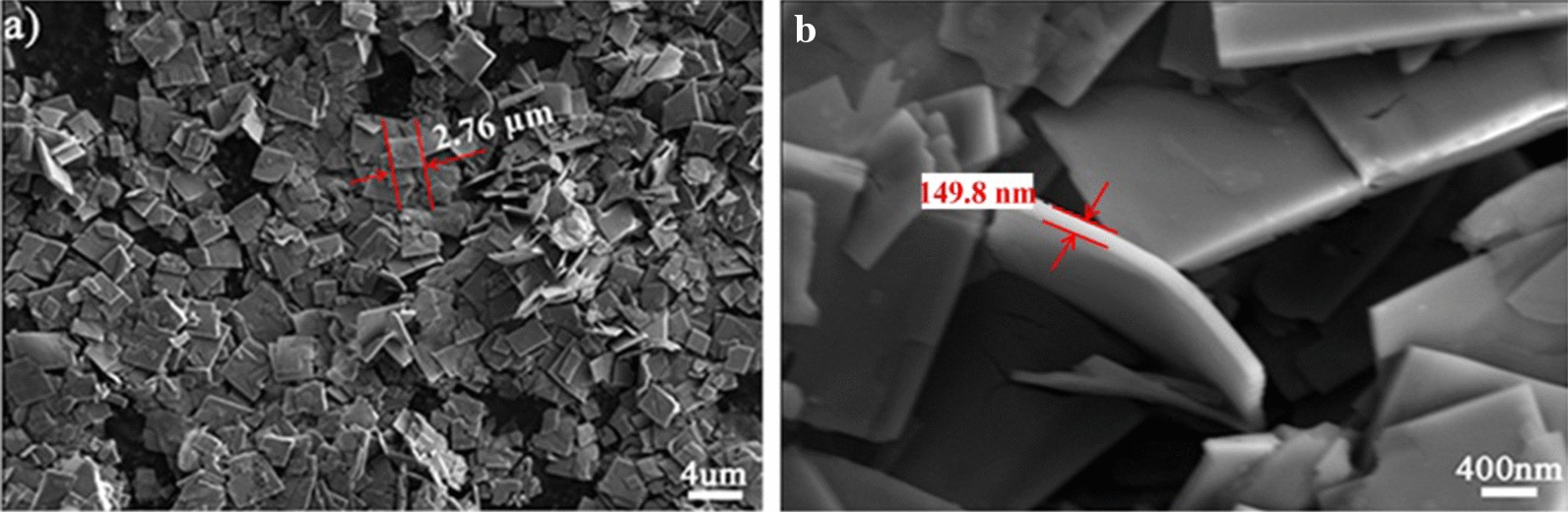
SEM images of EuCrMo_6_ microflakes

Figure 8 shows a typical SEM micrograph of the EuCrMo_6_ microflakes. From the SEM images, uniform flakes can be observed clearly in microsize. Each flake reveals a regular dimetric shape with the ca. 2.76 µm side length. From the literatures known so far, Keggin type POMs are always employed as building blocks to construct PNMs. Various POMs with different structures or components are seldom used in this research field. In this work, Anderson-type POM CrMo_6_ is used, hoping to generate new results. Fortunately, a rare flake-like PNMs is separated during this work. Therefore, it is expected to prepare more PNMs with interesting morphologies and properties by using diversified POM precursors.

**Fig. 9:**
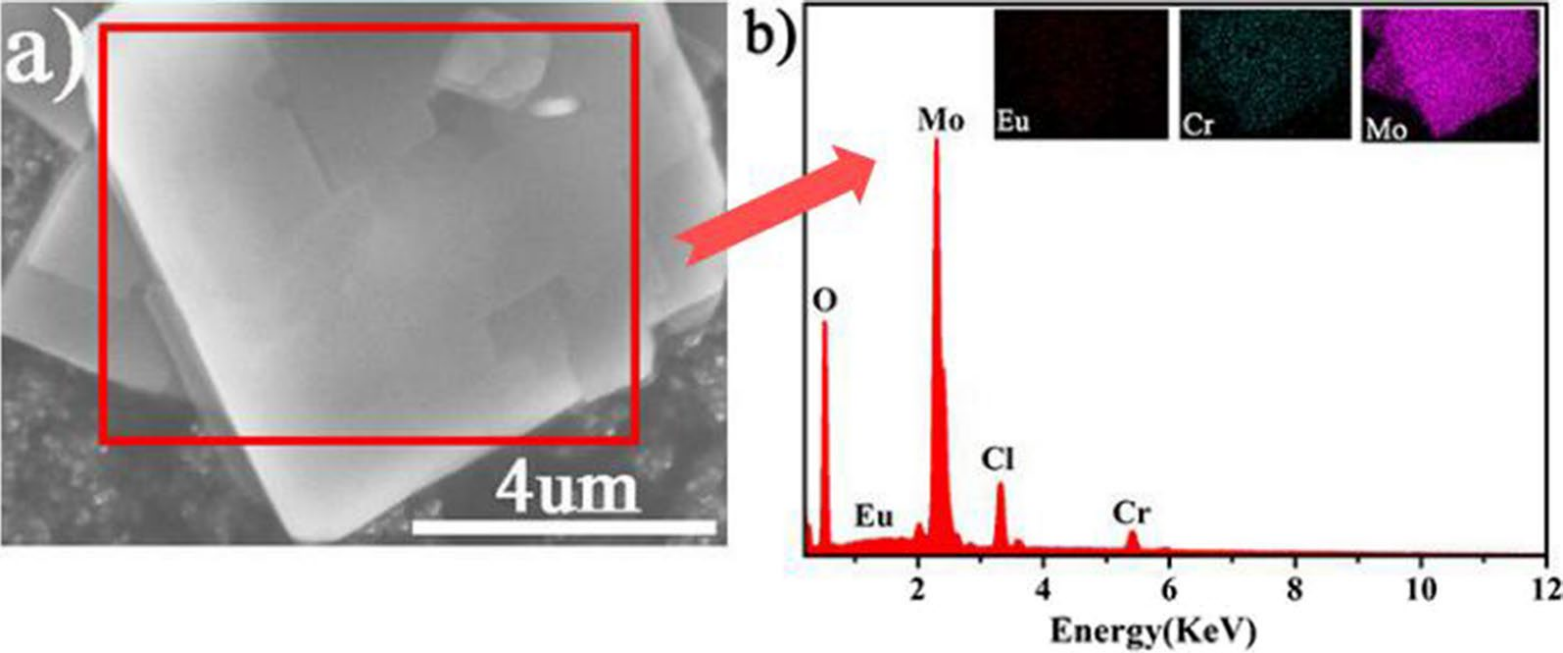
Corresponding element mappings and EDX of EuCrMo_6_ microflakes

Element mappings and EDX analysis for the microflakes was also recorded, which clearly exhibits the corresponding components of EuCrMo_6_ (Fig. 9). The analysis evidently proves the presence of Eu, Cr and Mo components. Meanwhile, the element mapping of Eu, Mo and Cr shows a homogeneous distribution in this composite.

### ICP-AES Results

Moreover, in order to accurately specify the contents of 3*d*–4*f* metals in each sample. ICP-AES experiments were performed on a Perkin-Elmer Optima 2100DV optical emission spectrometer to estimate the contents of Eu, Cr, Mo in EuCrMo_6_ and Ce, Cd, W in CeCdW_12_. In the first place, the results confirm the compositions of these materials, each sample contains 3*d*–4*f* metals. In the second place, it is worth pointing out that ICP-AES results are consistent with EDX results (Additional file 1: Fig. S1). In particular, these data could be used to conclude the atomic ratio of these materials. Integrating the results of IR, XRD, EDX and ICP-AES, the formulas K_6_[Ce(NO_3_)_3_]_3.5_CdCl_2_[H_2_W_12_O_40_]·19H_2_O and (NH_4_)_3_ [Eu(NO_3_)_3_]_0.005_[CrMo_6_O_24_H_6_]·11H_2_O is established for CeCdW_12_ nanoflowers and EuCrMo_6_ microflakes, respectively.

XPS spectra.

**Fig. 10:**
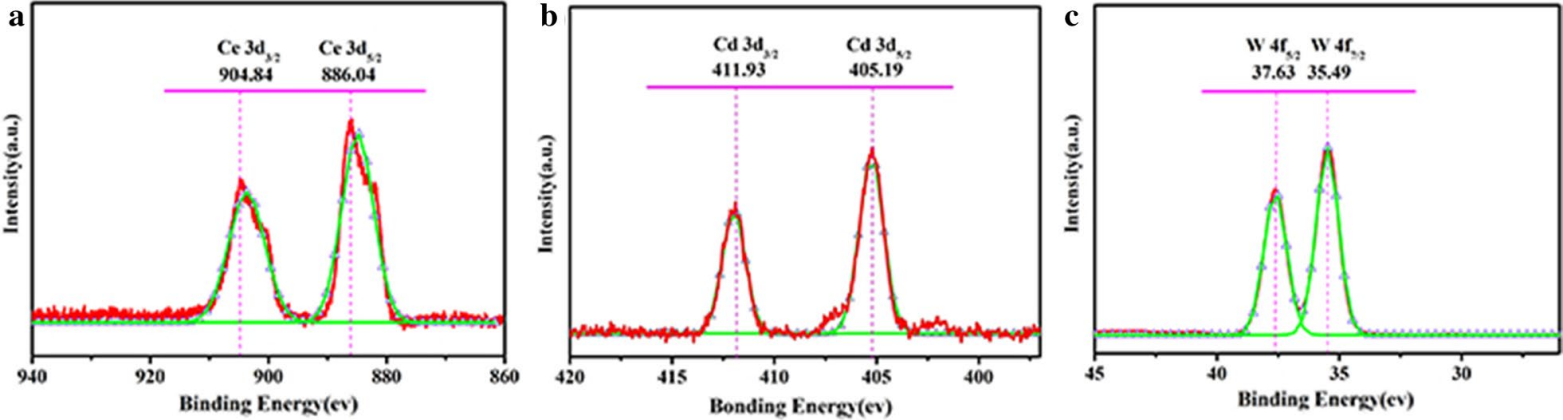
XPS spectra of CeCdW_12_ nanoflowers: **a** Ce 3*d*; **b** Cd 3*d*; **c** W 4*f*

**Fig. 11:**
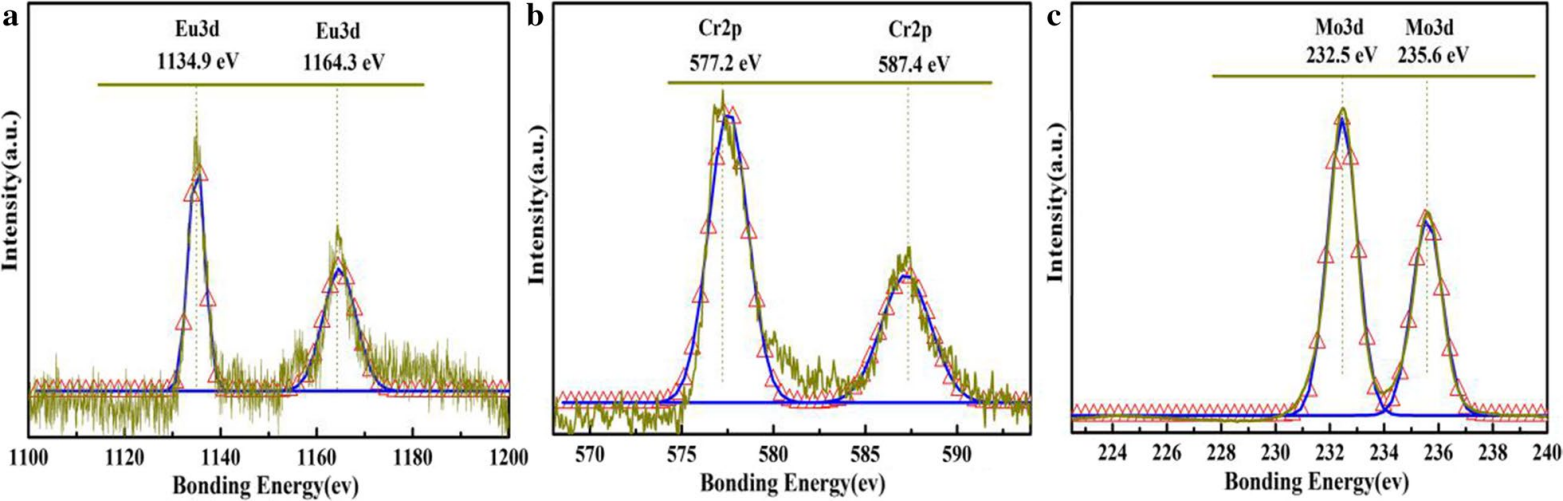
XPS spectra of EuCrMo_6_: **a** Eu3*d*; **b** Cr2*p*; **c** Mo3*d* (dark yellow line: experimental data; red scatter: fitting curve; blue line: spin–orbit partner lines)

The CeCdW_12_ nanoflowers were also characterized by XPS. Using a Shirley background subtraction, the fitting curves are shown in Fig. 10. The Ce3*d* shows a series of obvious signals in XPS spectrum. In particular, the strong satellites centered at 904.8 eV and 886.0 eV indicate the existence of Ce^3+^ ions [8]. The Cd3*d* spectrum exhibits two strong fitted peaks centered at 405.2 eV and 411.9 eV, proving the presence of Cd^2+^ ions [19]. The W4*f* spectrum exhibits two strong fitted peaks centered at 35.5 eV and 37.6 eV, which are attributed to the 4*f*_7/2_ and 4*f*_5/2_ spin orbit of W^6+^ ions in the isopolytungstate [28, 29], respectively. Additionally, the EuCrMo_6_ microflakes were also characterized by XPS. Using a Shirley background subtraction, the fitting curves are shown in Fig. 11. The Eu3*d* XPS peaks have a binding energy of 1134.9 eV and 1164.3 eV, indicating the Eu^3+^ ion is incorporated into microflakes and chelated to oxygen of CrMo_6_ (Fig. 11a). The peaks around 577.2 and 587.4 eV in the energy regions of Cr2*p* are confirmed to the Cr^3+^ centers in EuCrMo_6_ microflakes (Fig. 11b). The Mo3*d* spectrum exhibits two strong fitted peaks (BE = 232.5 eV, 235.6 eV) which correspond to the 3*d*_5/2_ and 3*d*_3/2_ spin–orbit of Mo^6+^ in the EuCrMo_6_ building block, respectively (Fig. 11c).

### ESI-MS Spectra (Negative Mode)

**Fig. 12:**
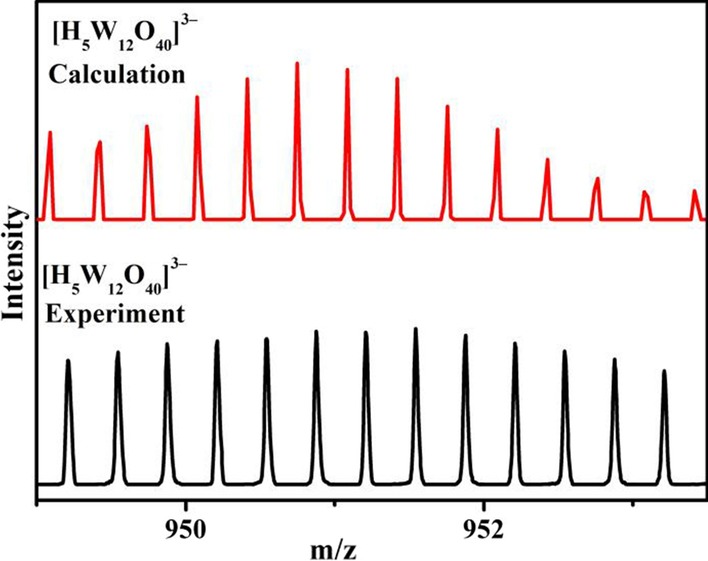
Negative mode ESI-MS spectra of CeCdW_12_ nanoflowers in distilled water in the range of 949–953.5 m/z

**Fig. 13:**
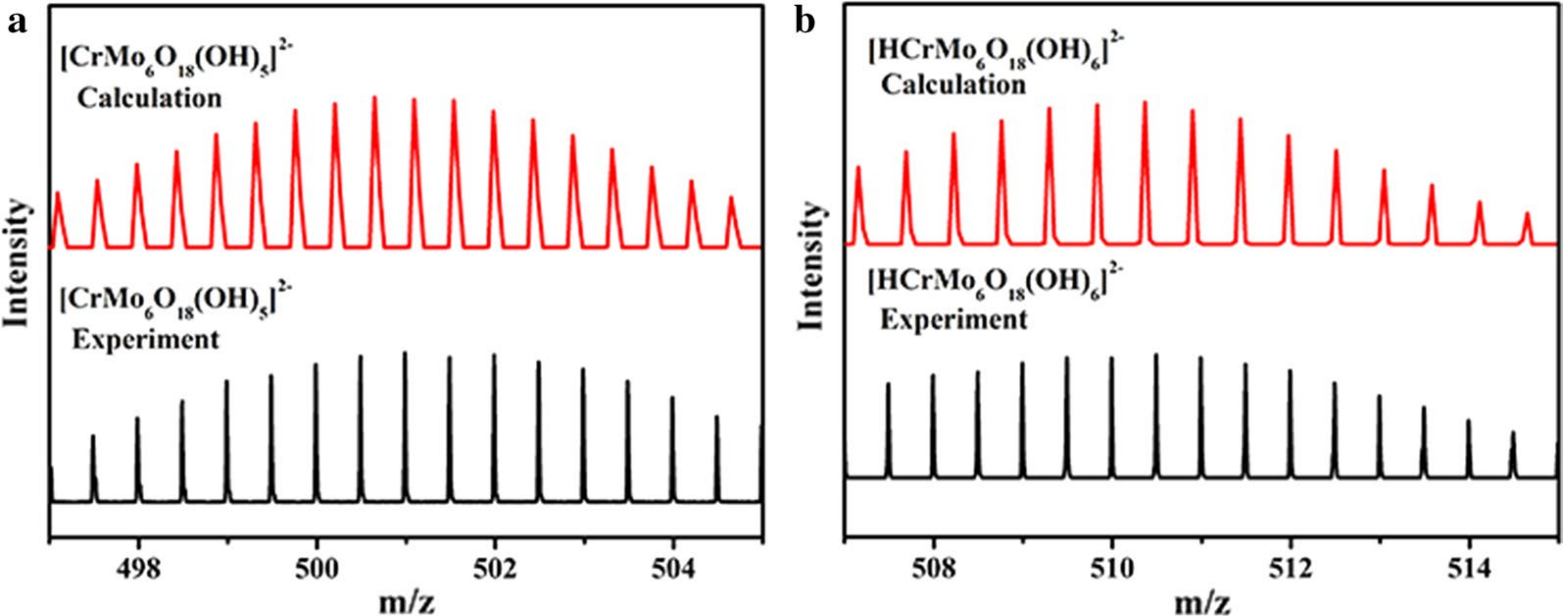
Negative mode ESI-MS spectra of EuCrMo_6_ microflakes in distilled water in the range of 865–887 m/z

The ESI-MS measurement has been found to be a useful analytic tool in studying the solution behavior of nano-sized clusters, which has been widely used to explore many types of POMs. Therefore, the ESI-MS spectra of CeCdW_12_ nanoflowers and EuCrMo_6_ microflakes in deionized water were performed in the negative ion mode, in order to confirm the identity of the clusters in the solution. As shown in Fig. 12, the signal appears at *m*/*z* = 950.2 attributed to the three charged anion [H_5_W_12_O_40_]^3–^, which shows CeCdW_12_ nanoflowers has some degree of stability in solution. As depicted in Fig. 13, a series of peaks (500.3 and 509.3 *m*/*z*) for − 2 charged ions are observed in the range of 495–515 *m*/*z*, which correspond to those peak positions for [CrMo_6_O_18_(OH)_5_]^2−^ and [HCrMo_6_O_18_(OH)_6_]^2−^, respectively. The results reveal that the Anderson type CrMo_6_ clusters retains their structural integrity in solution.

### Photoluminescence Property

**Fig. 14:**
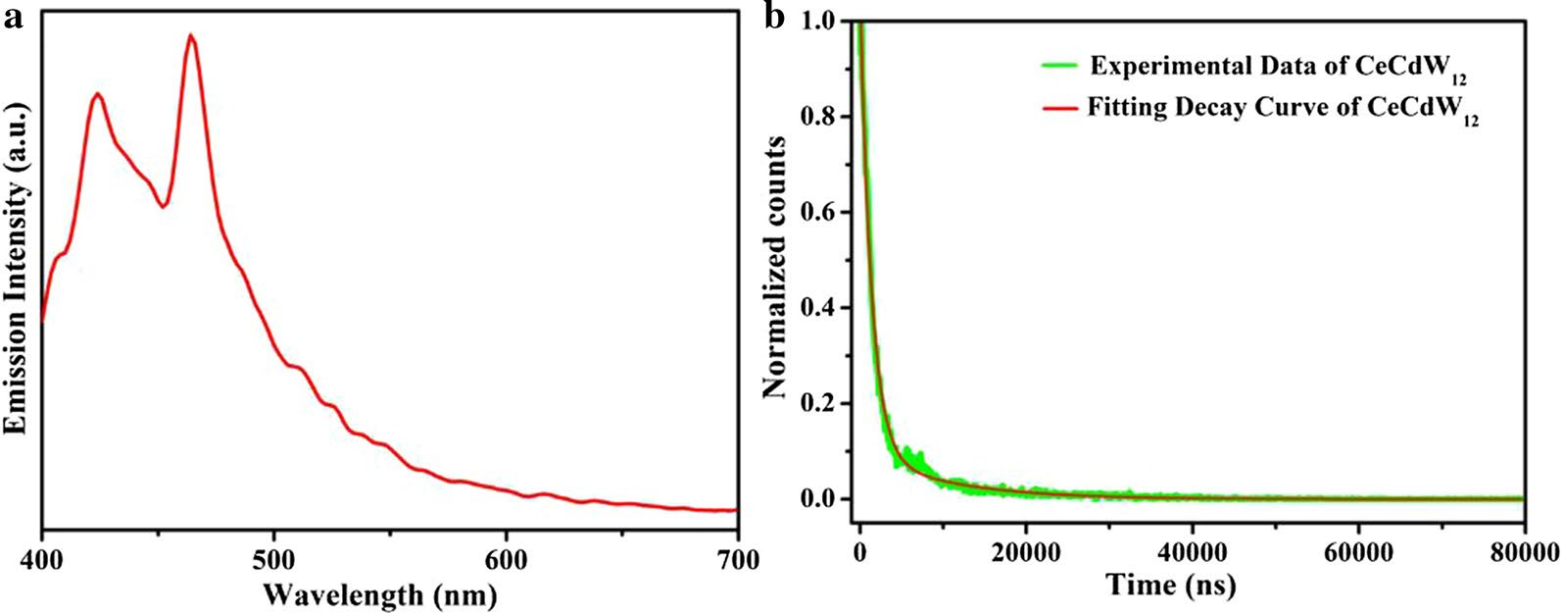
**a** Emission spectrum of CeCdW_12_; **b** PL decay curve of CeCdW_12_

**Fig. 15:**
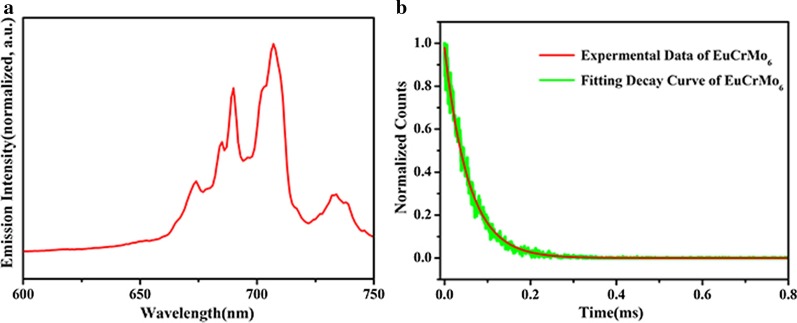
**a** Emission spectrum of EuCrMo_6_; **b** PL decay curve of EuCrMo_6_

The PL property of POM-based nano/micromaterials are still lacked of research which limits the functional applications in W-LEDs, luminescent thermometers, and temperature-dependent imaging reagents [30, 31]. In particular, the PL property of rare earth ions in isopolyoxometalate and Anderson type POM-based nano/micromaterials. In this work, CeCdW_12_ nanoflowers were utilized to explore the fluorescent behavior of Ce^3+^ ions. The samples were investigated in powders scattered on a plate intersecting with incidence at an angle of 45°. As depicted in Fig. 14a, upon excitation at 360 nm, the emission spectrum of CeCdW_12_ nanoflowers exhibits two peaks at 424 and 464 nm, corresponding to the Ce^3+^ ions related fluorescence. Besides, EuCrMo_6_ microflakes were utilized to explore the fluorescent behavior of Eu^3+^ ions. As depicted in Fig. 15a, upon excitation at 396 nm, the emission spectrum of EuCrMo_6_ displays five prominent *f* − *f* emitting peaks at 674, 685, 690, 707, and 734 nm that are assigned to Eu^3+ 5^D_0_ → ^7^F_J_ (*J* = 0, 1, 2, 3, 4) transitions [35]. It is worth to note that the strong PL peak of Eu^3+^ is at 707 nm in EuCrMo_6_ microflakes. This is interesting for in most cases the 618 nm is the strong peak. Various reasons may contribute to the phenomenon. Without doubt, the red shift of Eu^3+^ emission spectrum is originated from the structure differences between bulk and microsized PL material [33]. Besides, as the Eu^3+^ dopants were incorporated into the microflakes it caused the second phase to precipitate, so the change of coulomb attraction force the Eu^3+^ activator to experience different crystal field, and lead to the red shift on the emission spectrum [34].

Figures 14b and 15b shows the results of PL lifetime measurements of CeCdW_12_ nanoflowers and EuCrMo_6_ microflakes. The PL decay curves of CeCdW_12_ and EuCrMo_6_ are both well fitted to bi-exponential *I*(*t*) = *A*_1_ exp(− *t/τ*_1_) + *A*_2_ exp(− *t/τ*_2_) function, where *A*_1_, *A*_2_ and *τ*_1_, *τ*_2_ are the pre-exponential constant and the lifetime. The results and related parameters are illustrated in Table 2. According to the previous reports, the PL lifetime of Eu^3+^ is about 3 ms and ca. 200 µs in nanoparticles and traditional single-crystal compounds, respectively [35, 36]. In this work, the PL lifetime of Eu^3+^ is reduced to 1.14 µs, some reasons contribute to the changing of PL lifetime. Firstly, defect states would be created in EuCrMo_6_ microflakes. Secondly, Eu^3+^ ions and polyanions could be bonded with coordinated bond. Thirdly, concentration quenching may be occurred after doping procedure. All the reasons would induce non-radiative pathways, resulting in shortening of the PL lifetime [36] (Table 1).

**Table 1:** The ICP-AES and EDX data of CeCdW_12_ nanoflowers and EuCrMo_6_ microflakes

	CeCdW_12_ (wt%)			EuCrMo_6_ (wt%)	
	ICP-AES	EDX		ICP-AES	EDX
Ce	9.180	8.36	Eu	0.055	0.140
Cd	6.495	7.66	Cr	4.336	2.570
W	44.300	43.62	Mo	46.620	51.150

**Table 2:** The fitting parameters and PL lifetimes

Sample	*τ*_1_ (ns)	*τ*_2_ (ns)	*A*_1_	*A*_2_	*τ*_av_ (µs)
CeCdW_12_	1195.63	8692.14	0.189	0.891	8.48
EuCrMo_6_	5.94	187.43	1535.43	159.01	1.14

### Magnetic Property

**Fig. 16:**
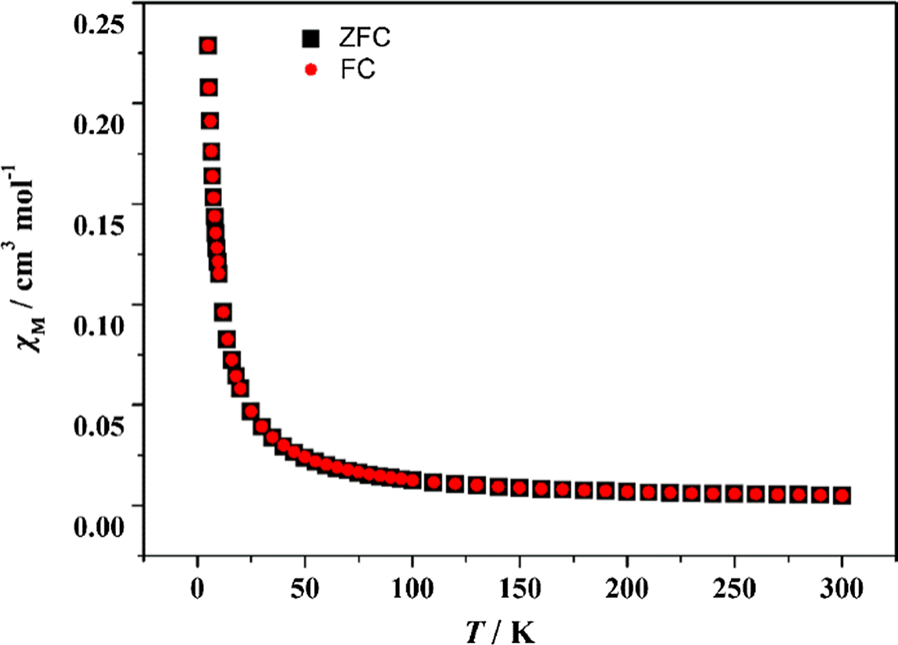
Temperature dependence of the ZFC and FC magnetization curves for EuCrMo_6_ in an applied field of 100 Oe

Bulk magnetization measurements were performed using a Quantum Design MPMS3 SQUID Magnetometer. The field sweep, as well as zero-field cooled and field cooled (ZFC/FC) magnetic susceptibility measurements from 5 to 300 K were performed on powder samples in gelatin capsules (Fig. 16). As shown in Fig. 16, ZFC curve and FC curve coincide, which manifests the presence of antiferromagnetic interaction.

**Fig. 17:**
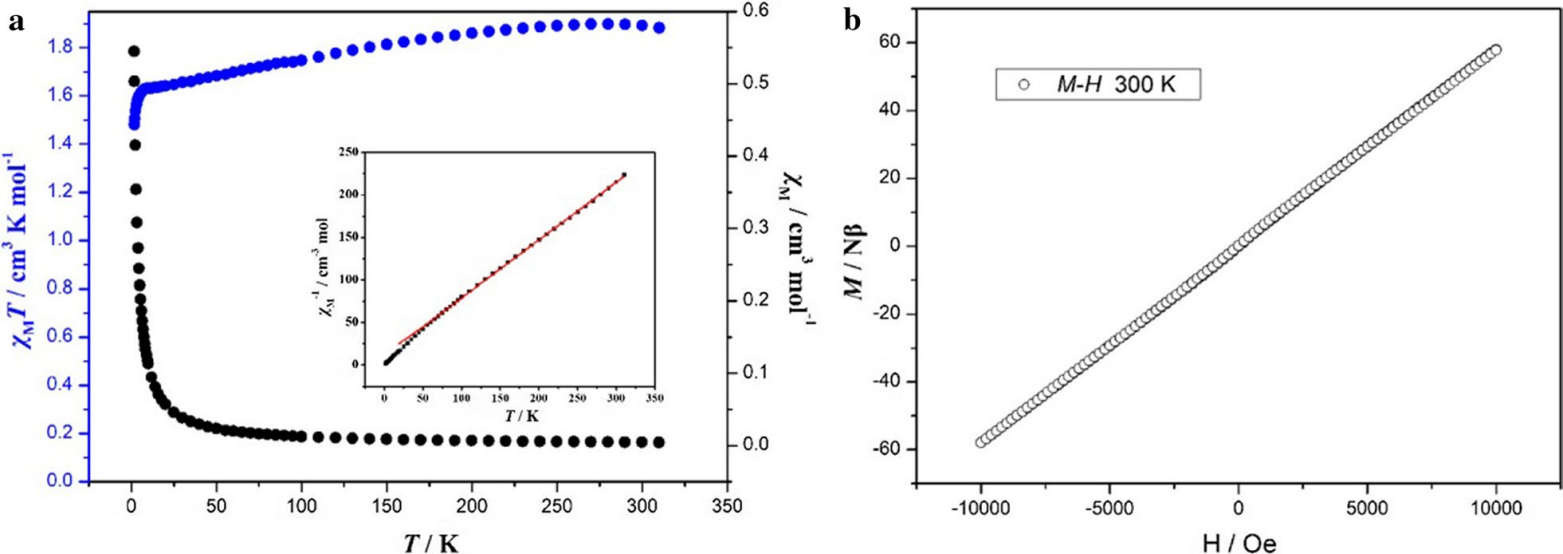
**a** 1/*χ* in the range of 1.8–300 K in 100 Oe for EuCrMo_6_. Red solid line corresponds to the best fit; **b** M–H curve at 300 K of EuCrMo_6_

As depicted in Fig. 17a, the *χ*_M_*T* value of EuCrMo_6_ at 300 K is 1.88 cm^3^ K mol^−1^, which is slightly lower than one isolated Cr^III^ ion (the experimental value is 1.98 cm^3^ K mol^−1^ calculated by Diaz et al***.*** with similar structural [LuCr]_*n*_ complex) [37].

As the temperature is lowered, the *χ*_M_*T* values gradually decrease up to a value of 1.63 cm^3^ K mol^−1^ at 8.0 K, and then sharply increase up to a maximum of 1.46 cm^3^ K mol^−1^ at 1.8 K, further indicating the existence of antiferromagnetic interaction. As shown in the illustration of Fig. 17a, curve fitting for 1/*χ* versus *T* plots of EuCrMo_6_ with Curie–Weiss law “*χ* = *C*/(*T *− *θ*)” in the range of 1.8–300 K results in *C* = 1.47 cm^3^ K mol^−1^, and *θ* = − 17.54 K. These results indicate that the Cr^3+^ ions reside in this formula and display anti-ferromagnetic interactions in low temperature, and the transition temperature is around − 17.54 K. Meanwhile, M–H curve of EuCrMo_6_ is recorded at 300 K (Fig. 17b). The result proves that the antiferromagnetic property at low temperature is transformed to paramagnetic property when the temperature increases to 300 K.

## Conclusions

In summary, CeCdW_12_ nanoflower and EuCrMo_6_ microflaky have been successfully prepared under mild solution conditions by introducing different 3*d*–4*f* metals. Unlike many other reported Keggin type PNMs, these materials are built from isopolyoxometalates or Anderson-type POMs. The combination of various 3*d*–4*f* metals and diversiform POMs not only enrich the components of PNMs, but also arise some unpredictable phenomena, such as the appearing of new morphology. Meanwhile, the existence of 3*d*–4*f* metals provides PNMs with multiple properties, for instance, photoluminescence, magnetism, catalysis and so on. In the following investigation, we will continue to investigate and explore the formation mechanism and the pertinent synthetic chemistry about 3*d*–4*f* metals doped PNMs.

## Supplementary information

**Additional file 1: Figure S1**. TG curves of CeCdW_12_ nanoflowers and EuCrMo_6_ microflakes.

## Data Availability

Data sharing is not applicable to this article as no datasets were generated or analyzed during the current study.

## Abbreviations

PNMs: Polyoxometalates-based nanomaterials
POMs: Polyoxometalates
CeCdW_12_: K_6_[Ce (NO_3_)_3_]_3.5_CdCl_2_[H_2_W_12_O_40_]·19H_2_O
EuCrMo_6_: (NH_4_)_3_ [Eu(NO_3_)_3_]_0.005_[CrMo_6_O_24_H_6_]·11H_2_O
SEM: Scanning electron microscope
EDX: Energy dispersive X-ray Spectroscopy
FTIR: Fourier transform infrared
XPS: X-ray photoelectron spectra
ICP-AES: Inductively coupled plasma optical emission spectroscopy
ESI-MS: Electrospray ionization mass spectrometry
PL: Photoluminescence
