# Supplementary material for: Controlled Synthesis and Properties of 3d–4f Metals Co-doped Polyoxometalates-Based Materials
Source: Nanoscale Res Lett. 2020 Nov 4;15:205. doi: 10.1186/s11671-020-03431-9 (PMC7642100; doi:10.1186/s11671-020-03431-9)
Supplement: Supplementary file 1 — Additional file 1: Figure S1. TG curves of CeCdW12 nanoflowers and EuCrMo6 microflakes. [file 11671_2020_3431_MOESM1_ESM.docx]

**Controlled Synthesis and Properties of** **3d-4f Metals Co-doped Polyoxometalates based Materials**

Ning Liu^a,†^, Ningning Guo^a,†^, Lin Sun^a^, Shixian Liu^a^, Guan Wang^a,^*, Yuan Zhao^b,^*

^a^Henan Key Laboratory of Polyoxometalate Chemistry, Institute of Molecular and Crystal Engineering, College of Chemistry and Chemical Engineering, Henan University, Kaifeng, Henan, China. E-mail: wangguan@henu.edu.cn

^b^The Key Laboratory of Natural Medicine and Immuno-Engineering, Henan University, Kaifeng, Henan, China. E-mail: zhaoyuan@henu.edu.cn

^†^Equal contributors

ORCID iDs: Guan Wang (https://orcid.org/0000-0002-0082-1875)

Yuan Zhao (https://orcid.org/0000-0001-6586-4350)


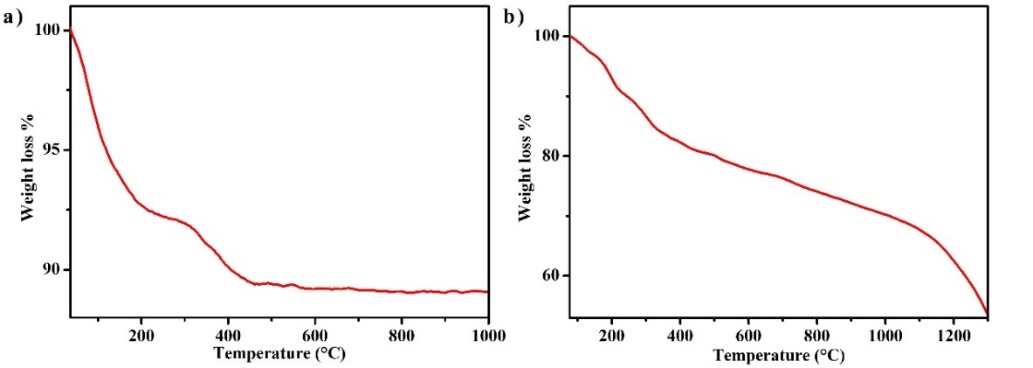


**Fig.S1** TG curves of CeCdW_12_ nanoflowers and EuCrMo_6_ microflakes.

Thermogravimetric (TG) experiments have been conducted to study the thermal stability of CeCdW_12_ nanoflowers and EuCrMo_6_ microflakes. The TG curves are shown in Fig. S1, which have been analyzed in nitrogen dynamic atmosphere at a heating rate of 10 ºC/min in the range of 35‒1000/1300 ºC and 9.970 and 9.149 mg samples are used in the form of powder, respectively. As depicted in Fig. S1a, the weight loss of CeCdW_12_ nanoflowers can be divided into two steps. The weight loss of 7.51% during the first step from 35 to 213 ºC involves the loss of adsorbed water molecules. On further heating, the material lose weight continuously during the second step with the weight loss of 3.09% from 213 to 1000 ºC, corresponding to the decomposion of POM molecules. As shown in Fig. S1b, the TG curve of EuCrMo_6_ microflakes gives a total weight loss of 46.60% in the range of 35‒1300 ºC. The weight loss of 15.83% during the first step from 35 to 339 ºC corresponds to the release of adsorbed water molecules. The second weight loss of 15.20% between 339‒1055 ºC is approximately attributed to the decomposion of POM molecules. The third weight loss of 15.57% from 1055 to 1300 ºC results from phase transitions of metal oxides.
